# Predictive models for one-year renal function improvement in patients with atherosclerotic renal artery stenosis (ARAS) following renal artery revascularization: a real-world study from China

**DOI:** 10.1080/0886022X.2026.2641914

**Published:** 2026-03-12

**Authors:** Lei Dong, Yuxi Li, Fangfang Fan, Jia Jia, Jianping Li

**Affiliations:** ^a^ Department of Cardiology, Peking University First Hospital, Beijing, China; ^b^ Institute of Cardiovascular Disease, Peking University First Hospital, Beijing, China

**Keywords:** atherosclerotic renal artery stenosis, renal artery revascularization, renal function, predictive models

## Abstract

The efficacy of interventional treatment for atherosclerotic renal artery stenosis (ARAS) in improving renal function remains controversial. The aim of this study was to develop a predictive model for estimating the probability of renal function response within one year following renal artery intervention in patients with ARAS. We retrospectively analyzed ARAS patients with renal artery intervention from January 2020 to December 2024 at Peking University First Hospital consecutively. Candidate predictors were selected using least absolute shrinkage and selection operator (LASSO) regression. A multivariable logistic regression model and a support vector machine (SVM) model were developed and internally validated using nested cross-validation. Discrimination was assessed by the area under the precision–recall curve (AUPRC), and calibration by the Brier score. 224 cases were enrolled, among which 47 patients exhibited renal function response following interventional treatment. LASSO regression identified four predictors (age, baseline renal function, diabetes, and bilateral renal artery stenosis) for model development. According to the results of internal validation, the mean AUPRC of logistic regression model was 0.701 (95% CI: 0.580, 0.834) and SVM model was 0.753 (95% CI: 0.642, 0.864). The logistic regression model demonstrated a mean Brier score of 0.150 (95% CI: 0.118, 0.182), whereas the SVM model with a mean Brier score of 0.148 (95% CI: 0.105, 0.193), indicating good calibration and stable performance. After further external validation, the models may serve as a risk stratification and post-procedural prognostic counseling aid for both patients and physicians.

**WHAT WAS KNOWN:**
Numerous previous studies have demonstrated that renal artery revascularization can improve or preserve renal function in certain patients with ARAS. However, there remains a lack of definitive criteria to guide patient selection for revascularization, and current evidence does not establish a clear consensus on which individuals are most likely to benefit from invasive intervention.

**THIS STUDY ADDS:**
Through this real-world study, we aim to evaluate the current clinical impact of interventional therapy on renal function in Chinese patients with ARAS and to develop a predictive model for estimating the probability of renal function response within one year following renal artery revascularization.

**POTENTIAL IMPACT:**
Our study developed and validated clinical prediction models to estimate the probability of renal function response in patients with ARAS within one year following renal artery revascularization. To a certain extent, these models may help support risk stratification and post-procedural prognostic counseling for both clinicians and patients after further external validation.

## 1. Introduction

Atherosclerotic renal artery stenosis (ARAS) constitutes a pivotal component of panvascular disease [1]. ARAS accounts for approximately 80% of renal artery stenosis cases and may lead to secondary hypertension and ischemic nephropathy [2]. In some instances, patients may progress to end-stage renal disease (ESRD) [3,4], imposing a substantial burden on both individuals and society. ARAS predominantly affects older adults who frequently present with traditional cardiovascular risk factors [5,6]. The primary pathogenic mechanism underlying ARAS is renal hypoperfusion, which subsequently activates the renin-angiotensin-aldosterone system (RAAS) [7,8]. Based on the pathophysiological characteristics of ARAS, it is evident that relieving renal artery obstruction and restoring adequate renal perfusion are critical therapeutic goals [9–11]. These interventions form the cornerstone of ARAS management.

However, the efficacy of interventional treatment for ARAS in terms of renal function improvement and patients’ long-term prognosis remains controversial. Several randomized controlled trials (RCTs) have failed to demonstrate a significant superiority of interventional treatment over medical therapy. Notably, the Cardiovascular Outcomes in Renal Atherosclerotic Lesions (CORAL) study, the largest RCT conducted to date, indicated that interventional treatment does not provide a substantial benefit for improving renal function in patients with ARAS [12]. A similar conclusion was reached in the ASTRAL study [13]. A meta-analysis of eight previous RCTs and multiple non-randomized comparative studies (NRCS) indicated that interventional treatment was not effective in reducing the incidence of ESRD [14]. However, several observational studies and clinical experience suggest that interventional treatment may confer significant benefits on renal function in specific patient populations [15–17]. Thus, a study is needed to address the following clinically relevant questions: Which patients are most likely to experience improvement in renal function following intervention? And, how can the potential for renal function improvement be systematically evaluated during preoperative discussions to adequately address the patient’s concerns? The aim of this real-world study was to investigate the current status of whether interventional therapy has a beneficial effect on renal function and develop a predictive model for estimating the probability of renal function response within one year following renal artery intervention in patients with ARAS.

## 2. Methods

### 2.1. Participants

**Figure 1.**
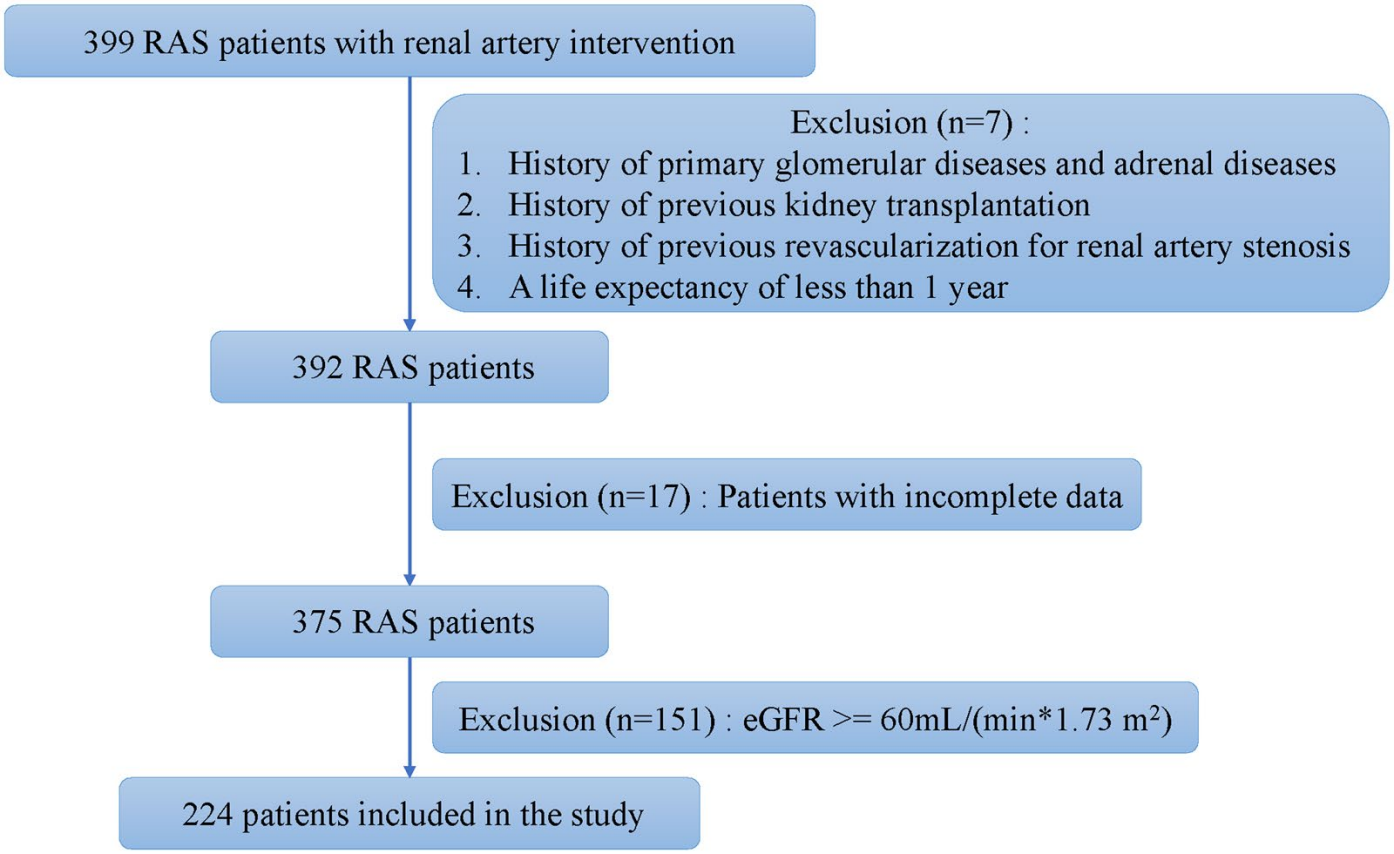
Study inclusion and exclusion flowchart.

This study consecutively included all patients with ARAS who underwent renal artery intervention and had renal insufficiency, at Peking University First Hospital from January 2020 to December 2024. The inclusion criteria were as follows: (1) Patients diagnosed with ARAS who received interventional treatment; (2) eGFR below 60 mL/(min·1.73 m^2^). The exclusion criteria included: (1) A history of primary glomerular diseases, adrenal disorders, or other conditions that may cause secondary hypertension and impair renal function; (2) A history of kidney transplantation; (3) Prior renal artery intervention; (4) Missing key baseline variables or unavailable renal function follow-up data within 12 months after the intervention. Patients with missing data in any variables required for the primary analysis/model were excluded prior to model development, and no imputation was performed. A study flow chart is illustrated in Figure 1.

### 2.2. Ethics declarations

This study was approved by the ethics committee of Peking University First Hospital (Approval No.: 2025R0649-0001). Given the retrospective nature of the study and the use of de-identified patient data with no direct risk to participants, the requirement for written informed consent was waived by the ethics committee. All procedures were conducted in accordance with the Declaration of Helsinki (2013 revision) and relevant national and institutional ethical guidelines.

### 2.3. Indications for intervention

Patients underwent renal artery intervention based on guideline-consistent clinical indications, including flash pulmonary edema, refractory (resistant) hypertension, rapid eGFR decline and bilateral severe stenosis, as documented in the medical record and institutional practice. Resistant hypertension was defined as blood pressure remaining above goal despite the use of ≥3 antihypertensive agents of different classes, including a diuretic, at maximally tolerated doses, or blood pressure controlled with ≥4 antihypertensive medications. Rapid eGFR decline was defined as *a* > 20% decrease in eGFR on repeat testing, exceeding expected biologic/measurement variability and warranting clinical evaluation.

### 2.4. Definition of the renal function response

A renal function response was defined as an increase in eGFR of more than 20% from baseline, with a final eGFR exceeding 15 mL/(min·1.73 m^2^) within 12 months after renal artery stenting, which is based on the American Heart Association Reporting Guidelines and prior publications [18]. Patients not meeting this definition were classified as non-responders, including those with unchanged or worsened renal function. Baseline characteristics were compared between responders and non-responders, and all prediction models were developed using this binary outcome.

### 2.5. Imaging assessment of stenosis

Renal artery stenosis was assessed using duplex ultrasound or magnetic resonance angiography (MRA) and digital subtraction angiography (DSA) in routine clinical practice. For MRA/DSA, stenosis severity was graded as the percentage luminal diameter reduction at the most narrowed segment relative to an adjacent normal reference segment. For duplex ultrasound, stenosis severity was assessed using standard Doppler criteria. All imaging studies were independently interpreted by two experienced readers; discrepant results were resolved by consensus (with a third adjudicator if needed). Image interpretation was performed as part of routine care and was not blinded to clinical information or post-intervention renal outcomes.

### 2.6. Data collection

In this study, we collected the clinical information about patients from a thorough review of relevant literatures and clinical judgment. Data were collected by trained research coordinators following standard operating procedures. These data included age, sex, duration of hypertension, types of antihypertensive drugs, history of diabetes, history of coronary artery disease (CAD), smoking history(defined as smoking 1 cigarette per day for at least half a year), systolic blood pressure (SBP), diastolic blood pressure (DBP), body mass index (BMI), degree of ARAS as determined by imaging, bilateral ARAS (defined as both renal arteries exhibiting stenosis greater than 50%), low-density lipoprotein cholesterol (LDL-C), brain natriuretic peptide(BNP), serum creatinine and eGFR at baseline and post intervention. Baseline laboratory values (including serum creatinine/eGFR and other baseline labs) were defined as the measurement closest to the time of intervention within 7 days prior to the procedure. Baseline blood pressure was defined as the measurement closest to the time of intervention within 48 h prior to the procedure (when multiple readings were available, the value closest to the intervention was used). The classification of chronic kidney disease (CKD) stages is based on the staging criteria introduced by the Kidney Disease: Improving Global Outcomes (KDIGO) organization, which has since become a globally accepted and widely implemented standard in clinical practice [19].

### 2.7. Development and evaluation of the predictive model

We performed nested cross-validation for model development and internal validation to reduce optimistic performance estimates. Outer and inner folds were stratified by outcome; outer held-out folds were used only for performance evaluation. Within each outer training set, an inner stratified 5-fold CV refit the entire pipeline, including least absolute shrinkage and selection operator (LASSO) with λ tuning (1-SE rule; λ. 1se) and model fitting/tuning. We developed two models: multivariable logistic regression and an RBF-kernel support vector machine (SVM). For SVM, continuous predictors were standardized using training-fold parameters only and applied to validation/test folds to prevent information leakage; C and γ were tuned in the inner loop on a log2 grid (C: 2^−5^ to 2^15^; γ: 2^−15^ to 2^3^). Hyperparameters were selected by grid search within the inner CV to maximize area under the precision–recall curve (AUPRC) (primary; given class imbalance), with area under the curve (AUC) as a secondary metric. Performance metrics (AUC, AUPRC, and Brier score) were calculated from outer out-of-fold predictions. We pre-specified logistic regression for interpretability and potential clinical translation, and SVM as a nonlinear comparator; more complex tree-based ensembles were not pursued given the limited event count and overfitting risk.

Calibration of the final refitted models was evaluated using calibration-in-the-large (intercept) and calibration slope, and visualized with bootstrap optimism-corrected calibration curves (*B* = 500 resamples).

After internal validation, we refitted LASSO on the full dataset to define a parsimonious final predictor set (four predictors) and refitted the final logistic regression and SVM models. A nomogram was constructed for the final logistic model. Receiver operating characteristic (ROC) curves from the refitted full-data models were presented as apparent performance for illustration, whereas the primary performance evidence was provided by the nested-CV out-of-fold estimates.

### 2.8. Statistical analysis

Data were summarized as mean (SD) for approximately normally distributed continuous variables and as median (interquartile range, IQR) for non-normally distributed continuous variables; categorical variables were summarized as n (%). Normality was assessed using visual inspection (histograms and Q–Q plots) and the Shapiro–Wilk test. Baseline characteristics were compared between the two groups using an independent-samples t-test for normally distributed continuous variables and the Wilcoxon rank-sum (Mann–Whitney U) test for non-normally distributed continuous variables. Categorical variables were compared using the χ^2^ test or Fisher’s exact test, as appropriate. All analyses were performed using R (version 4.5.1), and a two-sided P value < 0.05 was considered statistically significant.

## 3. Results

### 3.1. Baseline characteristics

A total of 224 eligible patients were included in this study. The median age of the patients was 67.0 (61.0 to 72.0) years; 67.0% were male, 34.8% had a diagnosis of diabetes mellitus and 25.4% had a CKD stage 4–5. The median duration of hypertension was 120.0 (48.0 to 240.0) months, and patients were, on average, taking 2.0 (1.0 to 3.0) antihypertensive medications. The median follow-up duration was 5.4 months, with no significant difference between the two groups. The distribution of patients’ clinical characteristics is shown in Table 1. Compared with unchanged/worsened group, patients in the renal function response group were more likely to younger, have a lower prevalence of diabetes and exhibit lower eGFR levels (all *p* < 0.05). There were no significant differences in other characteristics between the two groups.

**Table 1.** Baseline characteristics of study participants.

Characteristics	Overall (*N* = 224)	Unchanged/worsened renal function (*N* = 177)	Renal function response (*N* = 47)	*P*-value
Sex				1.000
Male	150 (67.0%)	119 (67.2%)	31 (66.0%)	
Female	74 (33.0%)	58 (32.8%)	16 (34.0%)	
Age (years), median (IQR)	67.0 (61.0 to 72.0)	68.0 (62.0 to 73.0)	66.0 (56.5 to 71.0)	0.028
Smoking history	129.0 (57.6%)	100 (56.5%)	29 (61.7%)	0.634
SBP (mmHg), median (IQR)	147.0 (133.0 to 160.2)	147.0 (133.0 to 159.0)	150.0 (134.0 to 165.0)	0.283
DBP (mmHg), median (IQR)	78.2 ± 13.9	77.3 ± 13.2	81.7 ± 15.9	0.052
Duration of hypertension (months), median (IQR)	120.0 (48.0 to 240.0)	120.0 (36.0 to 240.0)	120.0 (24.0 to 240.0)	0.586
Antihypertensive drugs	2.0 (1.0 to 3.0)	2.0 (1.0 to 3.0)	2.0 (1.0 to 3.0)	0.329
Diabetes	78.0 (34.8%)	69 (39.0%)	9 (19.1%)	0.018
CAD	57.0 (25.4%)	44 (24.9%)	13 (27.7%)	0.839
LDL-C (mmol/L), median (IQR)	2.1 (1.7 to 2.6)	2.1 (1.7 to 2.5)	2.1 (1.8 to 2.8)	0.201
BMI (kg/m^2^), median (IQR)	24.7 ± 3.1	24.7 ± 3.3	24.8 ± 2.6	0.756
BNP (pg/mL), median (IQR)	110.0 (49.0 to 190.0)	108.0 (49.0 to 187.0)	116.0 (50.0 to 266.5)	0.710
Degree of stenosis (%), median (IQR)	90.0 (80.0, 95.0)	90.0 (80.0 to 95.0)	90.0 (85.0 to 90.0)	0.784
Bilateral	108.0 (48.2%)	90 (50.8%)	18 (38.3%)	0.172
eGFR	41.3 (29.4 to 51.5)	44.0 (33.0 to 53.0)	33.1 (23.9 to 45.1)	<.001
Stage				0.041
CKD3a	89.0 (39.7%)	77.0 (43.5%)	12.0 (25.5%)	
CKD3b	78.0 (34.8%)	62.0 (35.0%)	16.0 (34.0%)	
CKD4	39.0 (17.4%)	26.0 (14.7%)	13.0 (27.7%)	
CKD5	18.0 (8.0%)	12.0 (6.8%)	6.0 (12.8%)	
Follow-up duration (months)	5.4 (3.6 to 8.0)	5.4 (3.4 to 8.0)	5.3 (3.0 to 7.8)	0.524

Abbreviation: eGFR, estimated glomerular filtration rate; CAD, coronary artery disease; SBP, systolic blood pressure; DBP, diastolic blood pressure; BMI, body mass index; LDL-C, low-density lipoprotein cholesterol; BNP, brain natriuretic peptide; CKD, chronic kidney disease; Statistical significance was assessed at *p* < 0.05.

### 3.2. Impact of interventional treatment on renal function in ARAS patients

Overall, the mean eGFR did not differ significantly between post-intervention and pre-intervention (41.3 [29.4 to 51.5] vs 40.7 [29.3 to 51.0] mL/(min·1.73 m^2^), *p* = 0.9844). Importantly, treatment response was heterogeneous: a subset of patients experienced postoperative eGFR improvement, and 19.6% (47/224) met the predefined renal function response criterion. These findings support the need to identify patients more likely to achieve meaningful renal functional improvement after stenting. In addition, 20 patients (8.9%) progressed to CKD stage 5 within 1 year after stenting, and there was no death.

### 3.3. Development and validation of predictive models

#### 3.3.1. Internal validation by nested cross-validation

Internal validation was performed using nested 10-fold cross-validation. All model-selection steps (LASSO-based predictor selection with λ tuning and SVM hyperparameter tuning) were carried out exclusively within the training data in each resampling iteration, and model performance was summarized from outer-fold out-of-fold predictions. In nested cross-validation, logistic regression achieved a mean AUPRC of 0.701 (95% CI: 0.580, 0.834), a mean AUC of 0.705 (95% CI: 0.595, 0.815), and a mean Brier score of 0.150 (95% CI: 0.118, 0.182). The SVM achieved a mean AUPRC of 0.753 (95% CI: 0.642, 0.864), a mean AUC of 0.762 (95% CI: 0.674, 0.849), and a mean Brier score of 0.148 (95% CI: 0.105, 0.193) (Table 2). Given class imbalance, AUPRC was prespecified as the primary discrimination metric, with AUC reported as a secondary measure.

**Table 2.** Discrimination and calibration performance of the models evaluated using nested 10-fold cross-validation.

	AUC	95% CI	AUPRC	95% CI	Brier score	95% CI
**Logistic regression model**						
	0.8666667		0.6986205		0.1234056	
	0.6838235		0.8788863		0.1516839	
	0.7555556		0.6434560		0.1342561	
	0.8058824		0.6179945		0.1549992	
	0.7708333		0.8305130		0.1083154	
	0.6666667		0.8098722		0.1420147	
	0.6111111		0.6469985		0.1768812	
	0.7013889		0.6833483		0.1424808	
	0.5833333		0.6078122		0.1628715	
	0.6055556		0.5876980		0.1990386	
Mean	0.7050817	(0.5948327, 0.8152179)	0.7005199	(0.5799512, 0.8335326)	0.1495947	(0.1176423, 0.1824876)
**SVM model**						
	0.6710526		0.7187926		0.1458972	
	0.7291667		0.7621376		0.2119484	
	0.7719298		0.7952181		0.1417924	
	0.8333333		0.7454821		0.1162797	
	0.7647059		0.5976386		0.1630869	
	0.7916667		0.8677212		0.1266001	
	0.7333333		0.7930876		0.1686141	
	0.9117647		0.6841832		0.1043841	
	0.6862745		0.7873424		0.1939501	
	0.7222222		0.7783954		0.1106877	
Mean	0.7615449	(0.6743815, 0.8489026)	0.7529998	(0.6423817, 0.8637195)	0.1483241	(0.1045831, 0.1927644)

Model performance was assessed within a nested cross-validation framework, in which the inner loop was used for model training and hyperparameter tuning and the outer loop was used for unbiased performance estimation.

Abbreviation: AUC, area under the receiver operating characteristic curve; AUPRC, area under the precision–recall curve; OR, odds ratio; CI, confidence interval.

#### 3.3.2. Calibration

**Figure 2.**
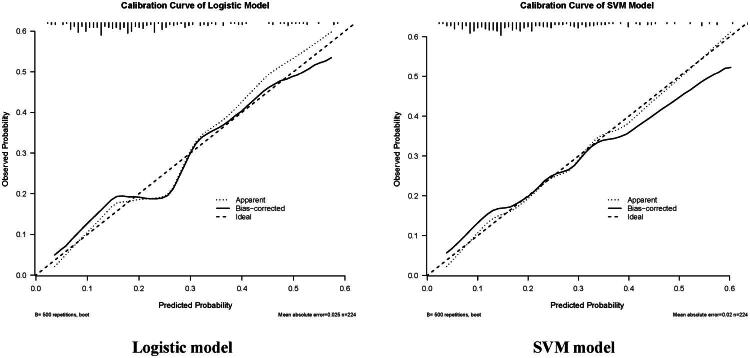
Calibration curve of the prediction models. The calibration plot compares the predicted probability of the outcome (x-axis) with the observed incidence (y-axis). The diagonal dashed line represents perfect agreement between prediction and observation, whereas the model curve indicates the actual calibration performance. A curve closer to the diagonal line suggests better calibration across risk strata.

Calibration of the final refitted models was assessed using bootstrap optimism-corrected calibration curves and calibration intercept/slope (Figure 2). For the logistic regression model, the calibration intercept was −0.158 (95% CI: −0.796, 0.462) and the calibration slope was 0.840 (95% CI: 0.411, 1.337). For the SVM model, the calibration intercept was −0.201 (95% CI: −0.801, 0.412) and the calibration slope was 0.811 (95% CI: 0.423, 1.292), supporting no major systematic over- or underestimation on average.

#### 3.3.3. Final predictor set (full-data LASSO for model specification)

**Figure 3.**
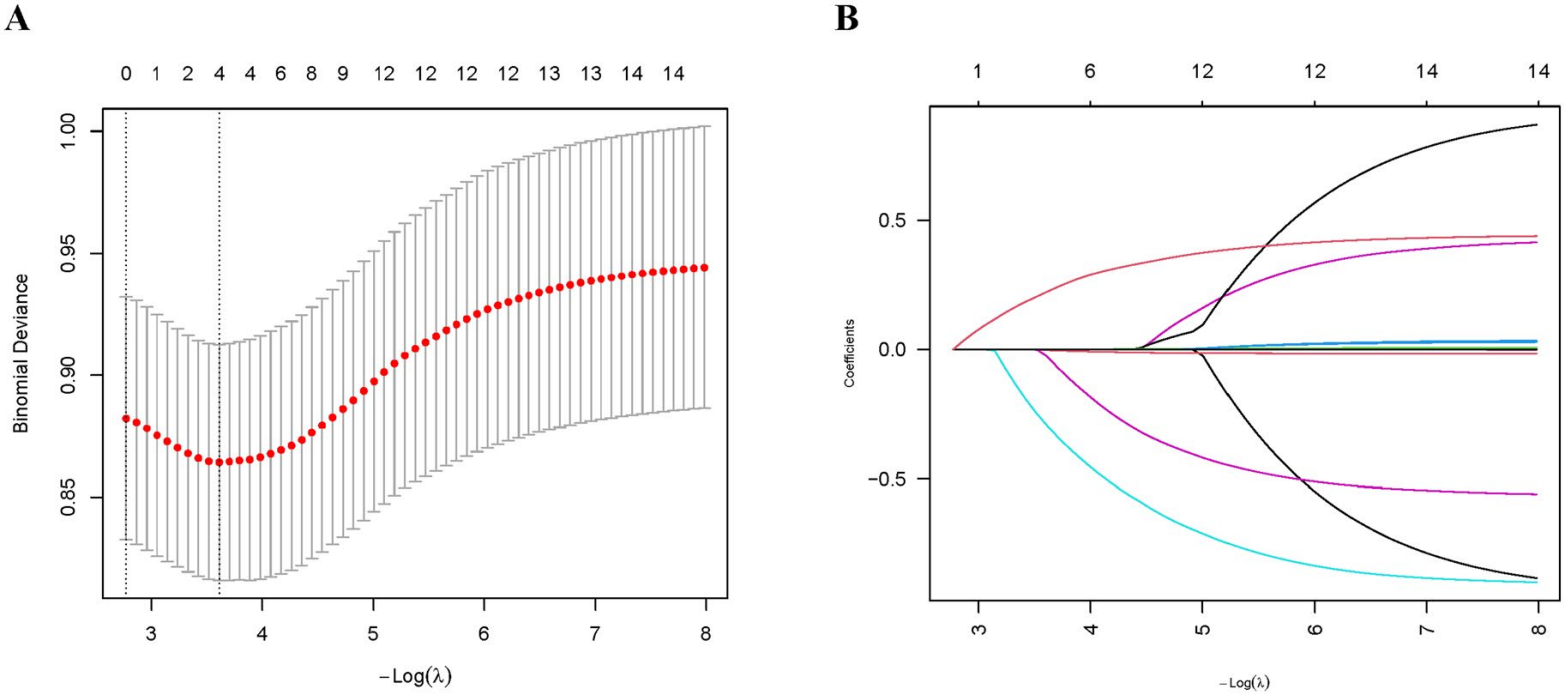
Screening of variables based on LASSO regression. (A) The cross-validation results. Four variables were selected when log(λ) = −3.64. (B) LASSO coefficient profiles of the 15 variables.

To define the final parsimonious model specification for reporting, we refit LASSO logistic regression on the full dataset and selected λ using the 1-SE rule (λ.1se). Four predictors were retained: age, baseline renal function (CKD stage), diabetes, and bilateral renal artery stenosis. The coefficient path and cross-validated deviance across λ values are shown in Figure 3.

#### 3.3.4. Full-data refit for graphical presentation

The results of the multivariate logistic regression analysis were shown in Table 3. The model formula of multivariable logistic regression model was:

logit (p)=log (p1−p)=1.39091+0.5766I3b+1.2326I4+1.1537I5−0.0408 age−1.0217 diabetes−0.5276 bilateral

**Table 3.** Candidate predictors included in the multivariable logistic regression model.

Predictors	OR	95% CI	*P*-value
Stage			
CKD3a	Ref.	Ref.	
CKD3b	1.78	(0.76, 4.24)	0.200
CKD4	3.43	(1.33, 9.03)	0.011
CKD5	3.17	(0.87, 11.0)	0.071
Age	0.96	(0.93, 0.99)	0.016
Diabetes	0.36	(0.15, 0.78)	0.013
Bilateral	0.59	(0.29, 1.19)	0.150

Abbreviation: CKD, chronic kidney disease; OR, odds ratio; CI, confidence interval. Statistical significance was assessed at *p* < 0.05.

**Figure 4.**
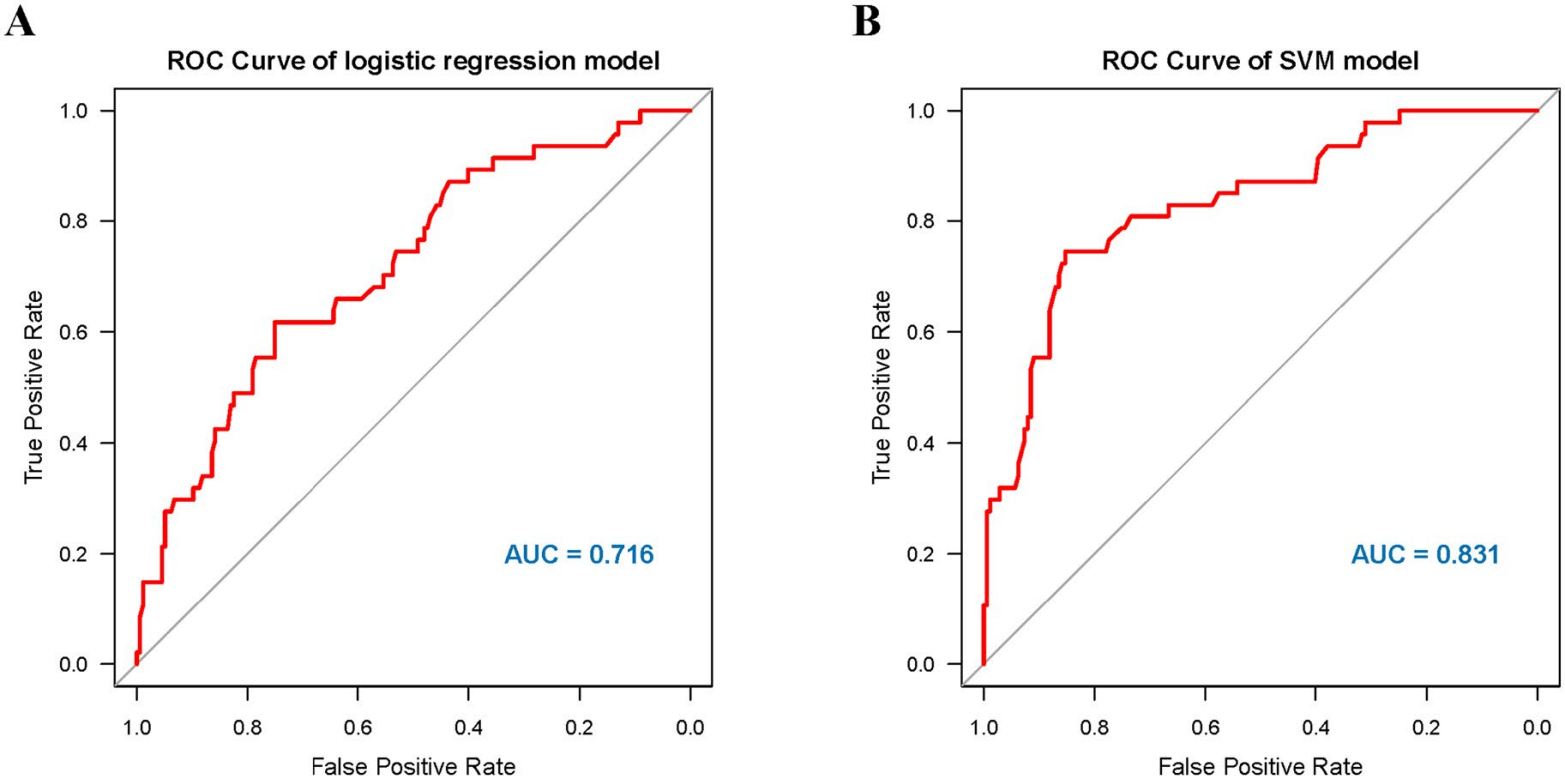
Receiver operating characteristic (ROC) curves and area under the curve (AUC) for model discrimination. ROC curves depict the tradeoff between sensitivity and 1 − specificity across classification thresholds. The AUC quantifies the overall ability of each model to discriminate between individuals with and without the outcome. (A) Logistic regression model; (B) support vector machine (SVM) model. Higher AUC values indicate better discriminative performance.

Following internal validation, we refitted both models on the full dataset using the final predictor set. ROC curves from these refitted models are presented as apparent performance for illustration (Figure 4). The apparent AUC was 0.716 (95% CI: 0.633,0.798) for logistic regression and 0.831 (95% CI: 0.763,0.899) for SVM, respectively. These plots are provided for visualization; the primary performance evidence remains the nested-CV out-of-fold estimates.

**Figure 5.**
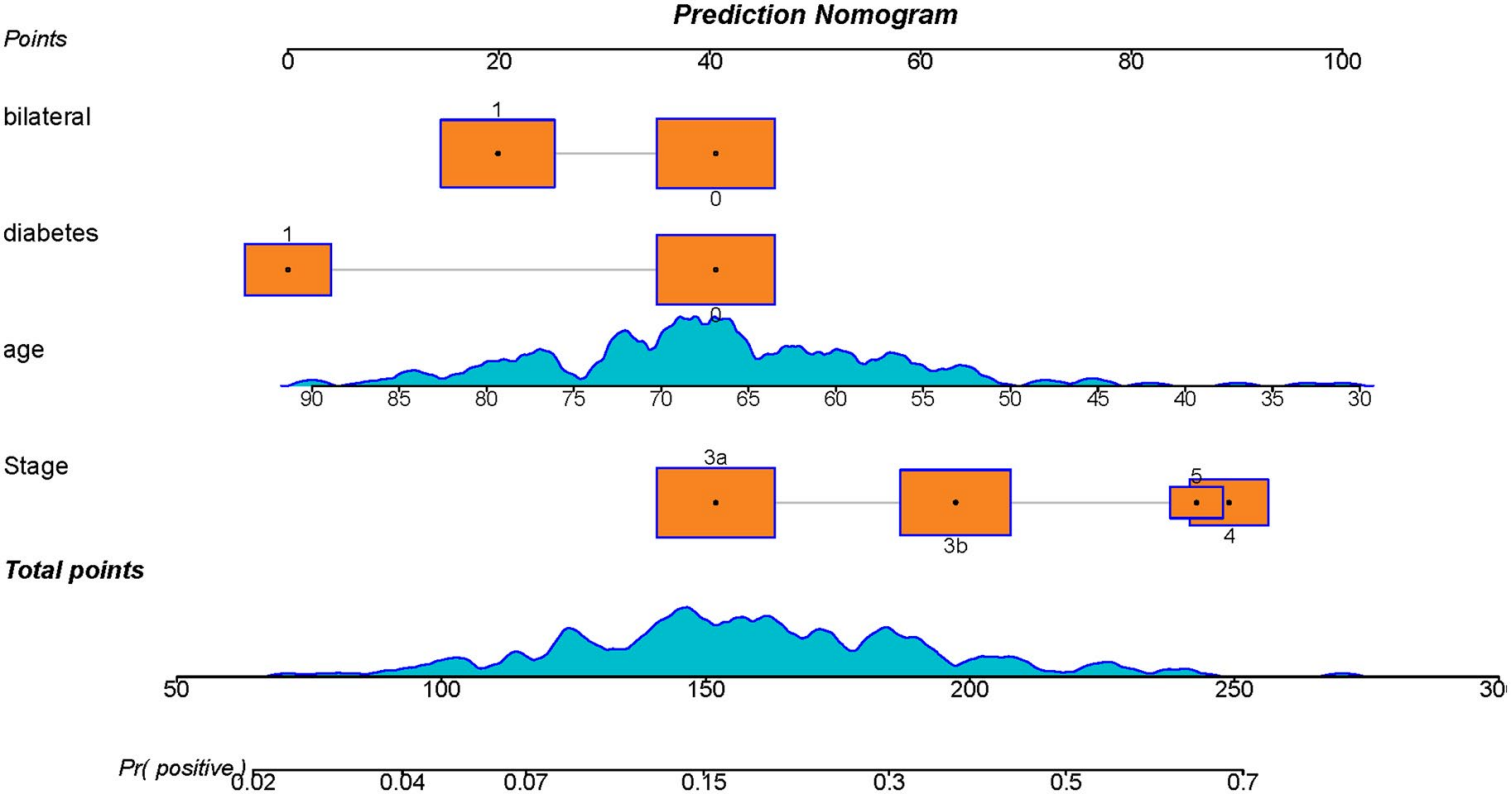
Nomogram for individualized risk estimation based on the logistic regression model. The nomogram enables individualized risk prediction and supports clinical decision-making.

A nomogram was constructed from the refitted final logistic regression model to facilitate individualized risk estimation (Figure 5). For each predictor, the nomogram assigns a point value that is proportional to its contribution to the linear predictor (i.e., the magnitude of the corresponding regression coefficient). The points from all predictors are summed to yield a Total Points value, which corresponds to the model’s linear predictor η. Finally, the nomogram maps Total Points to the predicted probability *p via* the logistic function, enabling direct readout of an individual’s estimated risk.

## 4. Discussion

In our clinical experience, only approximately one third to one-quarter of patients with ARAS exhibited improved renal function during late follow-up after interventional treatment [20–23]. This observation was generally consistent within our cohort. This outcome is concerning, given the limited tools and specific guidelines available to clinicians at the bedside for directing patient selection in ARAS management. This study integrated comprehensive clinical, imaging, and laboratory data from ARAS patients and established one-year prediction model of the renal function response following renal artery intervention. The models included four predictive factors: baseline CKD stage, diabetes history, age and bilateral ARAS. Renal function and diabetes history were strongly associated predictors, which was align with previous study [24,25]. Furthermore, our models provided an estimate of the likelihood of postoperative improvement in renal function by these commonly available and easily accessible clinical indicators, thereby assisting clinicians in addressing a key clinical concern for patients.

The renal function response to renal artery intervention has been controversial. The study by Anna Kabłak-Ziembicka et al. demonstrated that enhanced renal function was significantly linked to the following variables: a preprocedural serum creatinine level >122 µmol/l and eGFR >30 mL/min/1.73 m^2^. Conversely, an eGFR below 30 mL/min/1.73 m^2^ was associated with a decreased probability of achieving improved renal function [24]. Nonetheless, in another small study involving 88 patients who underwent renal artery stenting for ARAS, independent predictors of short-term improvement of renal function included eGFR and diabetes status. Patients with lower renal function are the most suitable candidates for interventional treatment [26]. The study conducted by Moutinho et al. reported that in patients with renal insufficiency, the improvement in renal function following interventional treatment was more pronounced in the subgroup with higher serum creatinine levels [27]. Moreover, Philip A Kalra et al.’s study revealed that percutaneous renal revascularization can improve renal function in advanced CKD (stage 4–5) [17]. In our cohort of Chinese patients, patients with CKD stage 3b, 4 and 5 were more likely to derive renal function response compared to those with CKD stage 3a. Notably, only patients with CKD stage 4 were significantly associated with renal functional response. These findings confirmed and extended results from prior studies.

Our study demonstrated that ARAS patients with older age and diabetes mellitus were less likely to benefit from interventional treatment. The study by Jia Fu et al. indicated that in patients who underwent renal artery stenting for ARAS, diabetes status was one of independent predictors of short-term improvement of renal function [26]. The study of Agnieszka Rosławiecka et al. indicated that ARAS patients who are older or have diabetes mellitus are at higher risk of developing renal failure [24]. These findings align with our study. The underlying mechanisms may be associated with glomerulosclerosis. Patients who are older and have diabetes are more likely to develop glomerulosclerosis [28–30]. This process may be related to the increased oxidative stress and the release of pro-inflammatory mediators [31]. It is evident that specific aspects of renal function in these patients have sustained irreversible impairment, and even the resolution of renal artery stenosis would not restore the compromised renal function. In addition, the study by Mark G. Davies et al. demonstrated that among patients who develop renal artery restenosis following interventional treatment, as many as 50% are diabetic [32]. Therefore, postoperative restenosis also contributes to the difficulty in renal function recovery in these patients.

A novel finding of this study was that patients with unilateral ARAS may be more likely to benefit from interventional treatment. In previous studies, the work of Haifeng Yu et al. had shown that GFR one month after stenting was associated negatively with baseline presence of bilateral ARAS [33]. These findings align with our study. In our study, bilateral ARAS was defined as both renal arteries exhibiting stenosis greater than 50%. The systemic atherosclerotic burden was considerable in these patients, which likely contributes to a poorer prognosis for renal function [34].

### 4.1. Strengths and limitations

Our study had some strengths. This study represented the first investigation of this research question within the Chinese patients. Several novel and noteworthy findings extended results from prior studies. More importantly, our investigation developed and validated predictive models for estimating the probability of renal function response in patients with ARAS following renal artery intervention. The indicators incorporated into the model are readily obtainable and can be applied even in regions with limited medical resources.

Although the results of the current study confirm and extend observations from prior studies, potential limitations of our study should be noted. First, model performance was assessed only by internal (nested cross-)validation in a retrospective single-center cohort, which may introduce selection bias and limits generalizability; therefore, external validation in independent cohorts—and ideally prospective, multicenter validation with impact evaluation—using more granular data on medical therapy, physiologic lesion assessment, and procedural characteristics is needed before any clinical implementation. Second, future work may explore more advanced machine-learning approaches (including deep learning) and leverage richer data sources to potentially improve predictive performance. Third, interpretability can be strengthened using established explainability methods and calibration-focused reporting. Overall, further validation and refinement in broader populations are needed to confirm generalizability and clinical utility.

## 5. Conclusion

Our study developed and internally validated a prototype clinical prediction model to estimate the probability of renal function response within one year among patients with ARAS undergoing renal artery intervention. Following external validation in independent cohorts, the models may enable risk stratification of these patients, thereby supporting post-procedural prognostic counseling in routine practice.

## Data Availability

The data that support the findings of this study are available on request from the corresponding author, Jianping Li. The data are not publicly available due to their containing information that could compromise the privacy of research participants.

## Abbreviations

ARAS: atherosclerotic renal artery stenosis
SVM: support vector machine
ESRD: end-stage renal disease
RAAS: renin-angiotensin-aldosterone system
RCTs: randomized controlled trials
NRCS: randomized comparative studies
eGFR: estimated glomerular filtration rate
CAD: coronary artery disease
SBP: systolic blood pressure
DBP: diastolic blood pressure
BMI: body mass index
LDL-C: low-density lipoprotein cholesterol
BNP: brain natriuretic peptide
CKD: chronic kidney disease
AUC: area under the curve
AUPRC: area under the precision–recall curve
LASSO: least absolute shrinkage and selection operator
